# Predictors of recovery from post-traumatic stress disorder after the dongting lake flood in China: a 13–14 year follow-up study

**DOI:** 10.1186/s12888-016-1097-x

**Published:** 2016-11-08

**Authors:** Wenjie Dai, Jieru Wang, Atipatsa C. Kaminga, Long Chen, Hongzhuan Tan, Zhiwei Lai, Jing Deng, Aizhong Liu

**Affiliations:** 1 Department of Epidemiology and Health Statistics, Xiangya School of Public Health, Central South University, Hunan, China; 2 Department of Pediatrics, University of Pittsburgh School of Medicine, Pittsburgh, USA; 3 Department of Mathematics, Mzuzu University, Mzuzu, Malawi; 4 Zhuhai Center for Disease Control and Prevention, Guangdong, China; 5 Hunan Provincial Center for Disease Control and Prevention, Hunan, China

**Keywords:** Post-traumatic stress disorder, Predictors, Recovery, Flood

## Abstract

**Background:**

Floods are some of the most common and destructive natural disasters in the world, potentially leading to both physical injuries and psychological disorders, including post-traumatic stress disorder (PTSD). PTSD can damage functional capacity and interfere with social functioning. However, little is known about recovery from PTSD after floods. This study used 2013–2014 follow-up data on survivors of the 1998 Dongting Lake flood who were diagnosed with PTSD in 2000 to measure the prevalence rate of PTSD at follow-up and identify predictors of recovery from the PTSD diagnosis in 2000.

**Methods:**

Participants included survivors who had been diagnosed as having PTSD in 2000 after the 1998 Dongting Lake flood. PTSD at follow-up was reassessed using the PTSD Checklist-Civilian version. Information on demographics, trauma-related stressors, post-trauma stressors, social support, and coping style were collected through face-to-face interviews. The association between the independent variables and PTSD at follow-up was analyzed using logistic regression analyses.

**Results:**

A total of 201 participants with a PTSD diagnosis in 2000 were included in this study. A total of 19.4 % of the flood survivors with PTSD in 2000 continued to suffer from PTSD in 2013–2014. In the multivariable logistic regression model, individuals who had lost relatives (OR = 12.37, 95 % CI = 2.46–62.16), suffered from bodily injury (OR = 5.01, 95 % CI = 1.92–13.08), had a low level of social support (OR = 5.47, 95 % CI = 1.07–27.80), or had a negative coping style (OR = 4.92, 95 % CI = 1.89–12.81) were less likely to recover from PTSD.

**Conclusions:**

The prevalence rate of PTSD at follow-up indicates that natural disasters such as floods may have a negative influence on survivors’ mental health for an extended period of time. Individuals who have lost relatives, suffered from bodily injury, had a low level of social support, or had a negative coping style were less likely to recover from PTSD. Therefore, effective psychological intervention measures are necessary for facilitating the recovery process from PTSD, especially for individuals with adverse prognostic factors.

## Background

Post-traumatic stress disorder (PTSD) is a psychological disorder caused by unusual threats or catastrophic events. According to the Fifth Edition of the Diagnostic and Statistical Manual of Mental Disorders (DSM-5), PTSD consists of four clusters of symptoms, namely, intrusion, avoidance, negative alterations in cognition and mood, and hyper-arousal [1]. PTSD can damage functional capacity and interfere with social functioning [2]. Hidalgo’s study showed that the lifetime prevalence of PTSD in the general population was approximately 1 to 9 % [3]. More generally, individuals with PTSD may experience a long recovery process after traumatic events [4]. For example, James found that half of the police officers with PTSD after the September 11, 2001 terrorist attacks—with a diagnosis made between 2003 and 2007 continued to have PTSD in 2011–2012 [5]. Additionally, according to a Chinese study, one-third of convalescent severe acute respiratory syndrome (SARS) patients with PTSD in 2003 were reported to have PTSD after the 4-year follow-up [6]. However, different traumatic events may result in different PTSD rates and different PTSD prognoses. Currently, research on PTSD has mainly focused on the incidence of PTSD or the risk factors of chronic PTSD after traumatic events such as earthquakes [7] and wars [8]. Studies on PTSD experienced after floods, let alone addressing the predictive factors of PTSD recovery after floods, are limited [9].

Floods are some of the most common and destructive natural disasters in the world, potentially leading to direct economic loss, death and psychological injuries, particularly in developing countries with limited coping mechanisms [10]. China has been seriously affected by floods. According to statistics, a flood in Sichuan in 2011 caused 31 deaths and 160 injuries [11]. Moreover, in 2010, a flood in Jilin damaged 301,000 houses, and a total of 118,000 houses collapsed [12]. The most devastating flood struck Dongting Lake in Hunan, China in 1998. This flood left hundreds of thousands of residents homeless and damaged many infrastructural and agricultural projects, leaving some survivors with psychological problems, including PTSD.

An epidemiological survey was conducted after the Dongting Lake flood between January and May 2000, revealing that the prevalence of PTSD among adult survivors was 9.2 % [13, 14], and the onset of PTSD after the flood was significantly associated with age, gender, education, flood experience, and social support [13, 15]. However, the prognosis of those individuals with PTSD was unknown. Therefore, the aim of our study was to investigate PTSD recovery progress and to identify likely predictive factors of PTSD recovery among Dongting Lake flood survivors who were diagnosed with PTSD in 2000.

## Methods

### Participants

Prior to this follow-up study, a cross-sectional study was conducted between January and May 2000, almost 2 years after the Dongting Lake flood. The study covered eight counties (Datonghu, Yueyang, Qianlianghu, Lingxiang, Huarong, Ziyang, Anxiang, and Longshan) that had been directly affected by the Dongting Lake flood in 1998. These eight counties are located in the south of the middle reach of the Yangzi River and form the catchment area of Dongting Lake. Survivors (aged 16 or above) of the disaster from these counties formed the target population. Diagnosis of PTSD was obtained by clinical interviews, and participants who were identified as having PTSD were recorded.

This follow-up study, therefore, considered the group of survivors diagnosed as having PTSD in 2000 in the previous study as the target population. In relation to the degree of destruction caused by the 1998 Dongting Lake flood, the eight affected counties were categorized as mild (Lingxiang and Longshan), moderate (Yueyang, Datonghu and Qianlianghu) and severe (Huarong, Ziyang and Anxiang). One county was randomly selected from each category to form the sampling frame, which consisted of Huarong, Yueyang, and Lingxiang counties. No additional floods had occurred in these three counties since the Dongting Lake flood in 1998. The three counties recorded 584 survivors with a PTSD diagnosis in 2000, and each survivor was considered for follow-up in this study. Excluded from this study were individuals (1) who could not express themselves normally, such as people with an intellectual disability, dementia, and/or a serious illness; (2) who had suffered from other types of intellectual disability or had taken any psychotropic drugs since the PTSD diagnosis in 2000; (3) who had received any psychological intervention since the PTSD diagnosis in 2000; and (4) who had incomplete data following data collection.

### Data collection

Qualified investigators who had either studied in a medical school or had worked for the local Centers for Disease Control and Prevention were appointed to collect data. Uniform training was given to the investigators, using a written investigation manual, before data collection began. Later, investigators conducted face-to-face interviews with the participants using a structured questionnaire to collect data regarding demographic characteristics, flood-related stressors, post-flood stressors, social support, and coping style and to ascertain PTSD symptoms. Each investigator received onsite supervision from professional psychologists. A total of 439 survivors with a PTSD diagnosis in 2000 were interviewed in Huarong in November 2013, and a total of 145 survivors with a PTSD diagnosis in 2000 were interviewed in Yueyang and Lingxiang in September 2014.

### Measures

#### Demographic variables

Gender, age, ethnicity, marital status, and education level were included in the analyses.

### Flood-related stressors

Following the protocol of most studies on natural disasters [16, 17], participants were asked the following questions to examine the intensity of the flood: Have you lost at least one family member? Have you or your family members been physically injured? Have you or your family lost most of your property? Have you or your family lost your livelihood? Have your homes been destroyed? These five questions were treated as dichotomous variables answered with either “Yes” or “No”.

### Post-flood stressors

Post-flood stressors, categorized as positive or negative in this study, were used to identify the stress situation in participants from the first investigation in the year 2000 to the present. Participants who suffered from post-flood stressors (e.g., traffic accidents, cancers, loss of a relative, etc.) and who reported feeling terrified (e.g., re-experiencing, avoidance and hyperarousal) were classified as positive for post-flood stressors.

### Social support

The Chinese version of the Social Support Rating Scale (SSRS) was used to assess the level of social support in this study. The Chinese version of the SSRS consists of three dimensions, namely, objective support, subjective support and support utilization. Collectively, the three dimensions have 10 items. The total score of these 10 items determined the level of social support of individuals at follow-up. A higher total score indicated better social support. The total score was classified as low (12–44), medium (45–54) or high (>55) according to the established guidelines. The Chinese version of SSRS has shown good reliability and validity [18].

### Coping style

The Simplified Coping Style Questionnaire (SCSQ), which consists of 2 subscales and 20 items for both subscales, was used to assess coping style in this study. The first subscale, positive coping, has 12 items referring to behaviours that actively buffer the stressful situation, such as “trying to find effective resolutions when faced with a stressful situation.” The second subscale, negative coping, has eight items referring to negative behaviours, such as “using intoxicating substances to get relief when faced with a stressful situation.” Each item is scored on a 4-point Likert scale, ranging from never (=0 points) to often (=3 points). A subscale score was calculated by averaging the scores of items for the subscale. A higher subscale score indicated more frequent use of the coping style in that subscale. Participants were classified as having positive coping if the positive coping style subscale score was higher than that of the negative coping style subscale. Otherwise, participants were classified as having negative coping. The SCSQ has demonstrated good reliability and validity with a test-retest reliability of 0.89 [19].

### PTSD

PTSD was identified by the PTSD Checklist-Civilian version (PCL-C), which was also used in the first investigation. The PCL-C was developed from the Fourth Edition of the Diagnostic and Statistical Manual of Mental Disorders (DSM-IV) and is a commonly used self-report questionnaire for identifying PTSD. According to some studies, the PCL-C has high internal consistency (α = 0.94) [20], with relatively high levels of sensitivity (94–97 %) and specificity (86–99 %) [21]. Moreover, research has shown that the Chinese version of the PCL-C has sound validity and reliability [22]. The PCL-C consists of 17 items that are split into three domains, namely, re-experiencing, avoidance and hyperarousal. Among the 17 items, all items referring to re-experiencing, half of the items referring to avoidance, and half of the items referring to hyperarousal contained event-specific wording (e.g., “…as a result of the Dongting Lake flood in 1998”). The scale of each of the 17 items ranges from 1 (not at all) to 5 (extremely). In this study, a score of 44 was used as a cut-off to identify PTSD at follow-up. The diagnostic efficiency of these criteria was 0.94 [21]. Participants were classified into two groups. The first group was the group that fulfilled the criteria for PTSD diagnosis during the follow-up period, while the second group was the group that met the diagnostic criteria for PTSD in the 2000 survey, but did not meet the diagnostic criteria for PTSD at the follow-up.

### Data analyses

Descriptive statistics were computed for the demographic variables, flood-related stressors, post-flood stressors, social support, and coping style. Univariable logistic regression analyses were used to identify the predictive factors of recovery from PTSD by consecutively exploring the roles of the preceding independent variables on recovery from PTSD. All of the statistically significant independent variables in the univariable logistic regression analyses were then used to perform multivariable logistic regression analyses to identify the independent predictive role of each variable on recovery from PTSD [23, 24]. The 95 % confidence intervals (95%CI) were provided for each odds ratio (OR). All tests were 2-tailed, and the significance level was set at 0.05. All analyses were performed using SPSS Version 19.0 (IBM Corp, Armonk, NY).

## Results

### Sample description

**Fig. 1:**
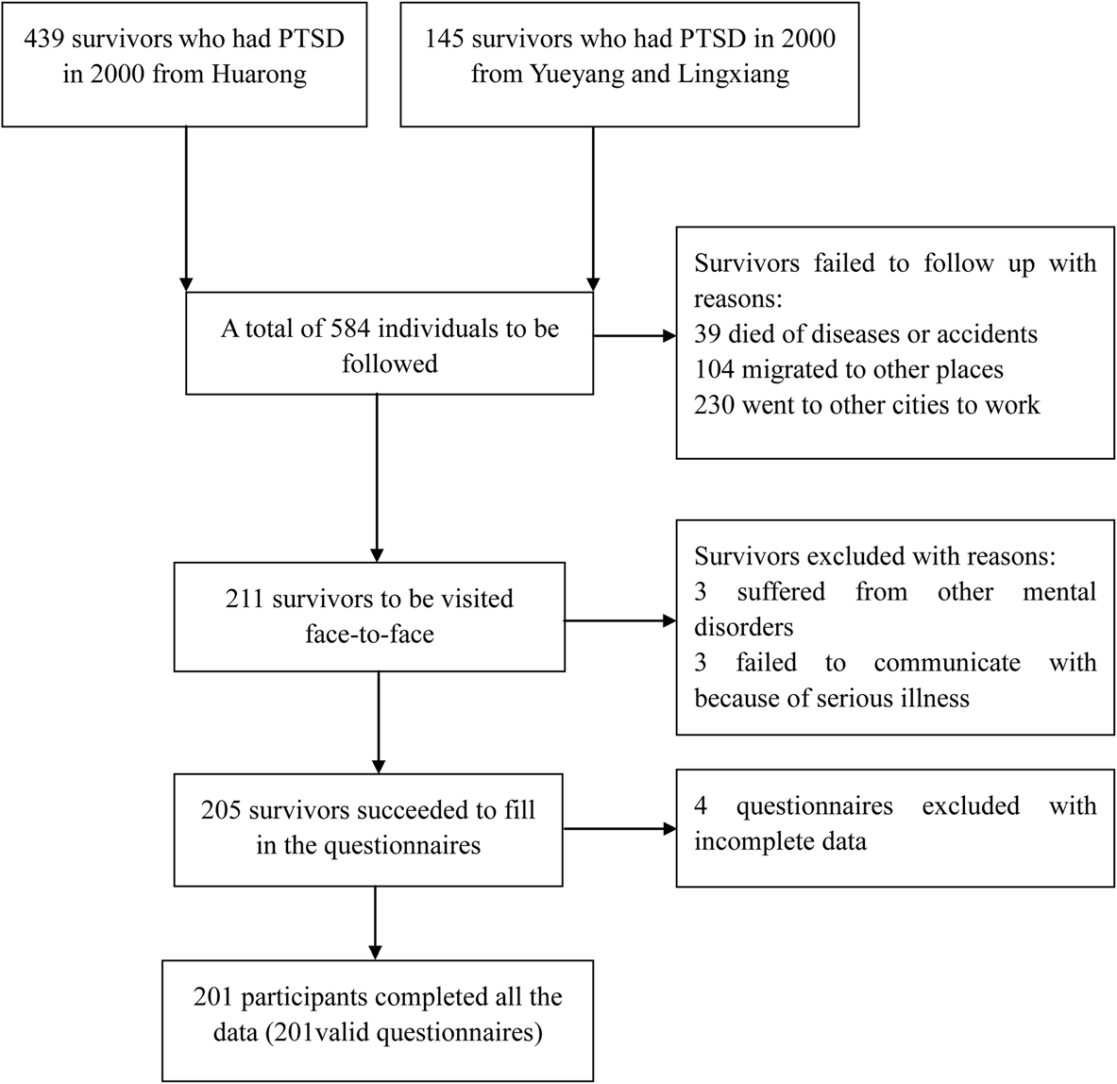
Flow chart of the participants included in this follow-up study. Presentation of how participants were enrolled in this study

A total of 584 survivors diagnosed with PTSD following the Dongting Lake flood in 1998 were identified. Of these potential candidates, 39 died of diseases or accidents, 104 migrated to other places, and 230 went to other cities to work. Thus, 211 survivors were contacted for interview representing an availability rate of 36.1 % (211/584) at follow-up. Of the 211 survivors contacted for interviews, 205 completed the questionnaires (Fig. 1). A total of 201 valid questionnaires were included in this study after excluding 4 incomplete questionnaires. A response rate of 98.0 % (201/205) was therefore achieved. Compared with those excluded due to non-response or incomplete data, those included in this study were much older (mean age on September 2014: 49 vs. 55 years, *P* < 0.05), but they had similar trauma exposure.

Descriptive data on demographics, flood-related stressors and post-flood stressors are presented in Table 1. Nearly half of the subjects were female. The mean (standard deviation) age of participants was 55 (12.02) years. Among the 201 participants, most were married, and all were of Han Ethnicity. Almost half had not received any education or had only attended primary school. In addition, nearly half of the participants had experienced loss of property or livelihood, and more than one third had been injured. Among all respondents, 13 had lost relatives in the flood. Post-flood trauma exposure was relatively low, with 78 % reporting no post-flood stressors.

**Table 1:** Characteristics of the participants (*n* = 201)

Variable		Number	Percent (%)
Gender			
	Female	90	44.8
	Male	111	55.2
Ethnicity	Han	201	100.0
Marital status			
	Married	182	90.5
	Unmarried	19	9.5
Age			
	30–60	142	70.6
	61–88	59	29.4
Education level			
	≤Primary school	106	52.7
	>Primary school	95	47.3
Loss of relative			
	No	188	93.5
	Yes	13	6.5
Bodily injury			
	No	129	64.2
	Yes	72	35.8
Loss of property			
	No	103	51.2
	Yes	98	48.8
Loss of livelihood			
	No	101	50.2
	yes	100	49.8
Damage to home			
	No	131	65.2
	Yes	70	34.9
Post-flood stressors			
	Negative	156	77.6
	Positive	45	22.4
Social support			
	Low	79	39.3
	Medium	80	39.8
	High	42	20.9
Coping style			
	Negative	54	26.9
	Positive	147	73.1

### Univariable analyses

Based on the PCL-C cut-off score of 44, the prevalence rate of PTSD at follow-up was 19.4 % (39/201). The results of univariable analyses indicated that males (OR = 0.49, 95 % CI = 0.24–0.99) were more likely to recover from PTSD in 2000 compared with females. Additionaly, participants with more serious exposure to flood trauma, such as loss of relatives (OR = 11.85, 95 % CI = 3.43–40.98), bodily injury (OR = 3.32, 95 % CI = 1.61–6.82), loss of property (OR = 3.35, 95 % CI = 1.56–7.18), loss of livelihood (OR = 2.07, 95 % CI = 1.01–4.27), or damage to home (OR = 2.36, 95 % CI = 1.16–4.80), were less likely to recover from their 2000 PTSD diagnosis. The univariable analyses also showed that individuals with low social support (OR = 8.57, 95 % CI = 1.92–38.36) or a negative coping style (OR = 6.08, 95 % CI = 2.87–12.84) were less likely to recover from their 2000 PTSD diagnosis (Table 2).

**Table 2:** Univariable logistic regression analyses of the effects of demographics, trauma exposure, social support, and coping style on the odds of PTSD at follow-up

	PTSD at follow-up			
Variable		Number (%)	OR (95 % CI)	*P*
Overall		39 (19.4)		
Gender	Female	23 (25.6)	1	
	Male	16 (14.4)	0.49 (0.24–0.99)	0.050
Marital status	Married	34 (18.7)	1	
	Unmarried	5 (26.3)	0.64 (0.22–1.91)	0.426
Age	21–60	29 (20.4)	1	
	61–88	10 (16.9)	0.79 (0.36–1.76)	0.571
Educational level	≤Primary school	24 (22.6)	1	
	>Primary school	15 (15.8)	0.64 (0.31–1.31)	0.222
Loss of relative	No	30 (16.0)	1	
	Yes	9 (69.2)	11.85 (3.43–40.98)	0.000
Bodily injury	No	16 (12.4)	1	
	Yes	23 (31.9)	3.32 (1.61–6.82)	0.001
Loss of property	No	11 (10.7)	1	
	Yes	28 (28.6)	3.35 (1.56–7.18)	0.002
Loss of livelihood	No	14 (13.9)	1	
	Yes	25 (25.0)	2.07 (1.01–4.27)	0.049
Damage to home	No	19 (14.5)	1	
	Yes	20 (28.6)	2.36 (1.16–4.80)	0.018
Post-flood stressors	Negative	28 (17.9)	1	
	Positive	11 (24.4)	1.48 (0.67–3.27)	0.334
Social support	High	2 (4.8)	1	
	Medium	24 (30.0)	3.94 (0.85–18.37)	0.081
	Low	13 (16.5)	8.57 (1.92–38.36)	0.005
Coping style	Positive	16 (10.9)	1	
	Negative	23 (42.6)	6.08 (2.87–12.84)	0.000

### Multivariable analyses

The results of multivariable logistic regression analyses are shown in Table 3. All variables that were statistically significant (*P* ≤ 0.05) in the univariable analyses were included in the multivariable model to identify the independent role of each predictor variable after adjustment for confounding. After multivariable analysis, gender, damage to home, loss of property, and loss of livelihood were no longer significantly associated with recovery from the 2000 PTSD diagnosis. The likelihood of PTSD at follow-up was higher for those who had lost relatives (OR = 12.37, 95 % CI = 2.46–62.16), suffered from bodily injury (OR = 5.01, 95 % CI = 1.92–13.08), had a lower level of social support (OR = 5.47, 95 % CI = 1.07–27.80), or had a negative coping style (OR = 4.92, 95 % CI = 1.89–12.81) than the respective reference groups.

**Table 3:** Multivariable logistic regression analyses of the factors significantly associated with PTSD at follow-up

Variable	B	SE	Wald	OR (95 % CI)	*P*
Loss of relative	2.515	0.824	9.327	12.37 (2.46–62.16)	0.002
Bodily injury	1.612	0.489	10.845	5.01 (1.92–13.08)	0.001
Low social support	1.698	0.830	4.188	5.47 (1.07–27.80)	0.041
Negative coping	1.593	0.488	10.651	4.92 (1.89–12.81)	0.001

## Discussion

The findings of this study, conducted over 15 years after the Dongting Lake flood in 1998, underscored the long-term impact of PTSD on survivors who were diagnosed with PTSD in 2000. It was found that the prevalence rate of PTSD among these survivors at the 13–14 year follow-up was 19.4 %. This prevalence rate emphasizes the importance of early identification of risk for long-term PTSD, which has been previously suggested [25–27]. Few studies have investigated PTSD in the long term or beyond 15 years [28, 29]. Among these studies, one showed that 7 % of Buffalo Creek flood survivors had a PTSD diagnosis at the 17-year follow-up [28]. In another study, 29 % of Aberfan disaster survivors met PTSD diagnostic criteria at the 33-year follow-up [29]. Additional research, with shorter longitudinal follow-up times, has found, for example, PTSD prevalence rates of 53 % among 9/11 police responders at the 5-year follow-up and PTSD prevalence rates of 34.3 % among SARS patients in 2000 at the 4-year follow-up [5, 6]. It is well-known that the type of traumatic event and the intensity of disasters may influence PTSD prevalence rates among survivors [15]. Moreover, follow-up time may also affect the PTSD prevalence rate. Longer follow-up time may provide survivors with sufficient time to recover from the trauma and, therefore, could result in a lower PTSD prevalence rate at follow-up.

Loss of relatives and physical injury inflict both physical and psychological pain in flood survivors. In this study, flood survivors who developed PTSD after the flood and had experienced loss of relatives or physical injury were less likely to recover from PTSD compared with their PTSD counterparts who did not experience loss of relatives or physical injury. In addition to this finding, a previous study showed that loss of relatives and physical injury were risk factors for the onset of PTSD after traumatic events [30]. Therefore, trauma-related stressors may not only be related to the onset of PTSD but may also be associated with PTSD recovery. Among the Chinese, for example, whether a family is cohesive plays an important role in one’s mental health, and kinship is the centre of the social network. Therefore, experiencing loss of relatives, especially a spouse or parents, may be particularly stressful for Chinese citizens. Additionally, the quality of life of individuals who have experienced bodily injury from the flood may be seriously affected by pain or disability brought about by the disaster. Therefore, it may be more difficult for them to recover from PTSD.

Social support refers to the quality and function of social relationships, and may have an effect on the way one copes with stress [31]. In this study, a high level of social support was found to be a predictor of PTSD recovery in flood survivors. This is in agreement with a previous study that found that social resources can provide a buffer against psychological distress following potentially traumatic events [32]. Moreover, social support also correlates with the onset of PTSD [33]. Our study found that social support was correlated with PTSD recovery. Therefore, it is important to provide more social resources to individuals who have experienced trauma, as these resources may have long-term impacts in alleviating the psychological effects caused by traumatic events.

Many previous studies have demonstrated that coping style is related to psychological outcome among individuals who have experienced trauma [34, 35]. Furthermore, Bonanno and his team found that deficits in coping flexibility were indicative of pathology in bereaved individuals [36]. Similar to their results, the present study found that coping style was significantly related to recovery from PTSD in flood survivors, and this is in support of the view that the ability to remain optimistic could be an effective way to cope with adverse events [37]. When faced with a trauma, individuals with a negative coping style were more likely to feel depressed, even hopeless [38, 39], which could negatively impact their recovery from mental illnesses.

With reference to previous studies, there have been contradictory results about the vulnerability of developing PTSD after traumatic events based on gender. Some studies have reported that females have a higher risk for PTSD symptoms [40–42], while other research has shown that males are more likely to develop PTSD symptoms after traumatic events [43]. The results of the present study showed that females were less likely to recover from prior PTSD in univariable analysis, but being female proved not to be an independent predictor of recovery from prior PTSD in the multivariabe analysis. Moreover, the effects of education level and age on the incidence of PTSD are controversial [40, 44, 45], and the results of this study indicated that neither variable was significantly related to PTSD recovery in flood survivors.

The role of stressful life events on the course of PTSD has been previously investigated [46]. For example, at follow-up 7–8 years after September 11, 2001 with the police officers who experienced the events, James found that 41.0 % had at least 2 life-threatening stressors since the terror attack [5]. In our study, 22.4 % of the participants reported post-flood stressors at a longer follow-up period than that in James’ study. This is mainly because the post-flood stressors indicated in this study included an assessment of the number of stressful life events and a rating of how terrified the person felt by each event. Thus, the rate of individuals who reported having both stressful life events (e.g., traffic accidents, cancers, loss of a relative, etc.) and feeling terrified (e.g., re-experiencing, avoidance and hyperarousal) may be decreased. However, this study found that post-flood stressors were not significantly associated with recovery from PTSD, although previous studies have indicated that post-disaster stressors or stressful life events were related to the psychological outcome of individuals following traumatic events [5, 46].

Despite having no significant correlation with PTSD recovery, it is worth noting that post-flood stressors play an important role in the course of PTSD. The reason for the lack of a significant relationship between them could be attributed to the fact that the post-flood stressors identified in this study were measured after a 13–14 year gap since the year 2000. This reasoning is supported by Perez, who found that the number of trauma exposures were a predictor of a worse course of PTSD, but only during some intervals in the 15-year follow-up period [47]. Therefore, it is possible that post-flood stressors and recovery from PTSD might be significantly correlated at the time when the participants were exposed to the post-flood stressors, but not when the follow-up study was conducted.

This study had some limitations that should be acknowledged. Firstly, the impact of income could not be analyzed because most of the participants did not want to provide information about their income. Secondly, fluctuations of PTSD symptoms over time were not assessed. Thirdly, participants who enrolled in this study were likely to have a chronic course since they were diagnosed with PTSD in 2000, almost 2 years after the Dongting Lake flood. Thus, the prevalence of PTSD at the 13–14 year follow-up may be overestimated. Finally, all participants of this study were Chinese of Han Ethnicity. Hence, the results may not be applicable to flood survivors in other populations.

## Conclusions

This follow-up study explored predictors of recovery from PTSD in flood survivors who experienced the 1998 Dongting Lake flood and were diagnosed with PTSD in 2000. The prevalence rate of PTSD among this group at the 13–14 year follow-up since the diagnosis of PTSD in 2000 was 19.4 %. Traumatic events such as floods may negatively affect survivors for a long period of time. Individuals who lost relatives due to the flood, suffered from bodily injury, had a low level of social support, or had a negative coping style after the flood were less likely to recover from PTSD. Therefore, with little or no psychological intervention following trauma, approximately one in five individuals diagnosed with PTSD at 2 years post-event will continue to experience severe PTSD symptoms, even 15 years later. More research is needed to design and evaluate early interventions following disasters, particularly for those with increased vulnerability.

## Abbreviations

95 % CI: 95 % confidence interval
DSM-5: Fifth edition of the diagnostic and statistical manual of mental disorders
DSM-IV: Fourth edition of the diagnostic and statistical manual of mental disorders
OR: Odds ratio
PCL-C: PTSD Checklist-Civilian version
PTSD: Post-traumatic stress disorder
SARS: Severe acute respiratory syndrome
SCSQ: Simplified coping style questionnaire
SSRS: Social support rating scale
